# Reduced surround suppression in monocular motion perception

**DOI:** 10.1167/jov.21.1.10

**Published:** 2021-01-15

**Authors:** Sandra Arranz-Paraíso, Jenny C. A. Read, Ignacio Serrano-Pedraza

**Affiliations:** 1 Faculty of Psychology, Universidad Complutense de Madrid, Madrid, Spain; 2 Biosciences Institute, Newcastle University, Newcastle upon Tyne, UK; 3 Faculty of Psychology, Universidad Complutense de Madrid, Madrid, Spain; 4 Biosciences Institute, Newcastle University, Newcastle upon Tyne, UK

**Keywords:** motion discrimination, visual suppression, binocular viewing, monocular viewing, sensory discrimination, surround suppression, motion suppression index, MT neurons

## Abstract

Motion discrimination of large stimuli is impaired at high contrast and short durations. This psychophysical result has been linked with the center-surround suppression found in neurons of area MT. Recent physiology results have shown that most frontoparallel MT cells respond more strongly to binocular than to monocular stimulation. Here we measured the surround suppression strength under binocular and monocular viewing. Thirty-nine participants took part in two experiments: (a) where the nonstimulated eye viewed a blank field of the same luminance (*n* = 8) and (b) where it was occluded with a patch (*n* = 31). In both experiments, we measured duration thresholds for small (1 deg diameter) and large (7 deg) drifting gratings of 1 cpd with 85% contrast. For each subject, a Motion Suppression Index (MSI) was computed by subtracting the duration thresholds in logarithmic units of the large minus the small stimulus. Results were similar in both experiments. Combining the MSI of both experiments, we found that the strength of suppression for binocular condition (MSI_binocular_ = 0.249 ± 0.126 log_10_ (ms)) is 1.79 times higher than under monocular viewing (MSI_monocular_ = 0.139 ± 0.137 log_10_ (ms)). This increase is too high to be explained by the higher perceived contrast of binocular stimuli and offers a new way of testing whether MT neurons account for surround suppression. Potentially, differences in surround suppression reported in clinical populations may reflect altered binocular processing.

## Introduction

The ability to discriminate correctly the direction of motion of a briefly presented stimulus depends on the contrast, the size, and the speed of the stimulus (Derrington & Goddard, 1989; Lappin et al., 2009; Tadin et al., 2003; see reviews about this topic in Nishida, 2011; Tadin, 2015). Increasing the size of a high-contrast moving Gabor patch increases the amount of time an observer needs to view it in order to correctly judge direction of motion (i.e., increases duration thresholds). This impaired performance with increased size and contrast is known as psychophysical surround suppression or spatial surround suppression (Lappin et al., 2009; Schallmo et al., 2018; Serrano-Pedraza, Hogg, & Read, 2011; Serrano-Pedraza et al., 2013; Tadin et al., 2003; Tadin & Lappin, 2005; Tadin et al., 2019).

The brain area most closely associated with motion perception is area MT/V5 of the middle temporal cortex. Although not all aspects of motion perception are reflected in MT (Alais et al., 1996; Ilg & Churan, 2004; Tailby, Majaj, & Movshon, 2010), psychophysical surround suppression does seem to be largely accounted for by the response properties of MT neurons with a center-surround antagonism (Allman, Miezin, & McGuinness, 1985a, 1985b; Born, 2000; Born & Tootell, 1992; Born et al., 2000; DeAngelis & Uka, 2003;; Liu et al., 2016, 2018; Tadin et al., 2011; Tanaka et al., 1986). For example, for brief presentation durations (40 ms), the firing rate of MT surround-suppressed neurons is reduced as stimulus size increases (Churan et al., 2008) and also for high contrasts (Pack, Hunter, & Born, 2005). On the contrary, it has been found that for low-contrast stimuli, direction discrimination is facilitated (i.e., duration thresholds are shorter) by increasing the size. This suggests spatial summation, that is, the signals presented by the stimulus at each location are summed over its entire extent, meaning that the output is larger for large stimuli (Serrano-Pedraza et al., 2011; Tadin et al., 2003). This result is also consistent with physiological data where MT neurons fire strongly for large stimuli at low contrast (Huang et al., 2008; Pack et al., 2005). Recent functional MRI (fMRI) studies have also found that neural responses in human MT complex (hMT+) reproduce perceptual suppression and summation (Er, Pamir, & Boyaci, 2020; Schallmo et al., 2018). Other psychophysical results have shown that the suppressive center-surround interactions increase with increasing speed (Lappin et al., 2009), results that are also consistent with physiological results that show that surround suppression of MT neurons depends on speed (Allman et al., 1985a; Pack et al., 2005; Tanaka et al., 1986; see also Born & Bradley, 2005).

In many of these previous studies, it has been assumed that the antagonistic surround is spatially symmetric around the excitatory center, and similarly, models of center-surround organization assume that receptive fields are circularly symmetric (Huang et al., 2008; Tadin et al., 2019; Tadin & Lappin, 2005). However, in a psychophysical experiment, Serrano-Pedraza et al. (2011) showed that surround suppression is stronger along the direction of motion, suggesting a spatial nonhomogeneity of the antagonistic surround. This result was in agreement with previous physiology results, where 80% of MT neurons presented a nonhomogeneity of the inhibitory surround spatial organization where one or two inhibitory regions were located on one side or on opposite sides of the excitatory receptive field and in both cases along the direction of motion (Raiguel et al., 1995; Xiao et al., 1995; Xiao et al., 1997; see also Born & Bradley, 2005). All these results suggest that psychophysical surround suppression is linked with physiological center-surround suppression in area MT (see a deeper review in Tadin, 2015).

MT neurons are binocular, this is, they are driven by stimuli presented to either eye (Czuba et al., 2014; DeAngelis & Uka, 2003; Maunsell & Van Essen, 1983; Tailby et al., 2010; Zeki, 1974). In a recent study, Czuba et al. (2014) have shown that most frontoparallel MT units showed the most balanced responses to motion in the two eyes, that is, they responded in a similar way to inputs from either eye. However, frontoparallel MT cells showed stronger responses when both eyes were stimulated than for monocular stimulation. If the strength of the response is different for monocular and binocular stimulation, the strength of surround suppression could also be different for each viewing condition.

Measuring the depth of binocular rivalry suppression, it has been found that monocular and binocular moving surrounds were both effective in increasing suppression depth of the target drifting in the same and opposite direction (Paffen, Alais, & Verstraten, 2005). Greater suppression depth has been found when the central rival target and surround moved in the same direction compared to when the surround moved in the opposite direction. However, they did not find significant differences between monocular and binocular surround conditions. Here, we compare surround suppression under monocular and binocular viewing conditions in the same observers, measuring duration thresholds. We quantify surround suppression using the Motion Suppression Index (Tadin et al., 2006) in two experiments with different techniques to achieve monocular viewing (mirror stereoscope vs. eyepatch). Our results for both techniques show that surround suppression is substantially stronger (1.79 times higher) under binocular viewing than under monocular viewing. These results could be consistent with a reduction of perceived contrast under monocular viewing (Campbell & Green, 1965; Legge, 1984). However, this factor of 1.79 is far larger than can be explained by existing data for high-contrast stimuli.

## Materials and methods

### Participants

In this research, we have performed two experiments. Eight volunteers, 3 males and 5 females ranging from 20 to 27 years old (mean ± SD, 22.37 ± 2.87 years), took part in the first experiment, and 31 volunteers, 9 males and 22 females ranging from 18 to 33 years old (mean ± SD, 22.45 ± 4.45 years), took part in the second experiment. Participants were unaware of the purpose of the study. The age criteria for participants were to be older than 18 years and younger than 35 years, given that motion surround suppression decreases with aging (Betts, Taylor, Sekuler, & Benett, 2005; Tadin & Blake, 2005; Yazdani et al., 2015), and to have normal or corrected-to-normal vision. We tested stereoscopic vision with the Frisby stereotest (at 40 cm) and the visual acuity in each eye with the ETDRS 2000 series visual acuity chart (at two distances, 40 cm and 300 cm) of the participants of Experiment 2. The average stereoacuity was 26.77 ± 13.45 arcsec (mean ± SD, *n* = 31, maximum stereoacuity value was 70 arcsec). The visual acuity averaged across both eyes (the highest difference between eyes was 0.1 logMAR) was 0.011 ± 0.09 logMAR (mean ± SD, *n* = 31) for a 40-cm distance (maximum visual acuity value was 0.25 logMAR) and –0.06 ± 0.095 logMAR (mean ± SD, *n* = 31) for a 300-cm distance (maximum visual acuity value was 0.25 logMAR). Experimental procedures were approved by the Complutense University of Madrid Ethics Committee and comply with the Code of Ethics of the World Medical Association (Declaration of Helsinki).

### Apparatus

In both experiments, the stimuli were presented on a gamma-corrected 19-in. Eizo Flex Scan T765 CRT monitor with a resolution of 1,024 × 768 pixels (horizontal × vertical) with a vertical frame rate of 120 Hz and mean luminance of 35.4 cd/m^2^. The luminance of the monitor was corrected using a Minolta LS-110 photometer (Konica Minolta Optics, Inc., Osaka, Japan). The observation distance to the stimuli was 70 cm. A chin rest (UHCOTech HeadSpot, Houston, TX, USA) was used to control the observation distance and to stabilize the head of the participants. Responses were recorded using the ResponsePixx Handheld (VPixx Technologies, Inc., Saint-Bruno, Canada). A computer Mac Pro 3.7 GHz Quad-Core Intel Xeon E5 (graphics card AMD FirePro D300 2048 MB) was used to control the experiments. In Experiment 1, the participants observed the monitor through a mirror stereoscope constructed with two beam splitters and two mirrors (Elliot Scientific Ltd, Harpenden, UK). In Experiment 2, we used orthoptic eyepatches (Opticlude; 3M, New York, USA) to occlude one eye during the sessions.

### Stimuli

**Figure 1.**
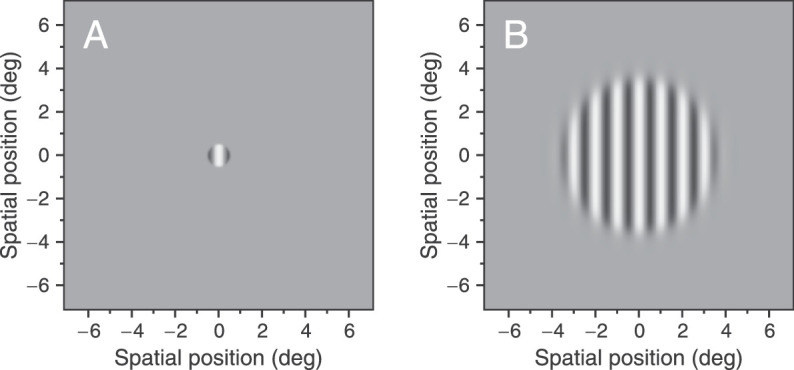
Examples of the stimuli used in the experiments. (**A**) Vertical grating of 1 c/deg windowed by a two-dimensional isotropic Butterworth function of 1 deg diameter. (**B**) Vertical grating of 1 c/deg with Butterworth window of 7 deg diameter. The Michelson contrast of these examples is 0.85.

The stimuli were programmed in MATLAB (The MathWorks, Natick, MA, USA) using the Psychophysics Toolbox extensions (Brainard, 1997; Kleiner, Brainand, & Pelli, 2007; Pelli, 1997) with 15 bits of gray-scale resolution (DataPixx Lite; VPixx Technologies, Inc.). The stimuli were vertical gratings of 512 × 512 pixels multiplied by a two-dimensional Butterworth spatial window of order 10 (González & Winz, 1987; see the equation for the low-pass filter in the appendix of Sierra-Vázquez et al. (2006), which was adapted to the spatial domain). This window is similar in shape to the window used in Schallmo et al. (2018) to measure duration thresholds. We tested two diameters of the Butterworth window (1 and 7 deg, referred to as “small” and “large” in the remainder of the article). We used this window instead of a previously used Gaussian window (Arranz-Paraíso & Serrano-Pedraza, 2018) because the size of the stimuli for Experiment 1 was physically limited by the screen size (i.e., half of the screen was used to present the stimuli in each eye). Thus, a Gaussian window with the same diameter would exceed the limits of the screen. All gratings had a Michelson contrast of 0.85 and a spatial frequency of 1 c/deg and drifted horizontally at a speed of 2 deg/s (see Figures 1A and 1B). In Experiment 1, the stimuli were presented in a square of 17.5 × 17.5 cm subtending a visual angle of 14.25 × 14.25 deg at ±7.13 deg of eccentricity and were observed through a mirror stereoscope (left eye only could view the left side of the monitor and right eye, the right side). If the stimulus was presented to the left eye, the right eye was stimulated with a uniform stimulus with the same mean luminance and vice versa. In Experiment 2, the stimuli were presented in the center of the screen. The contrast of the stimuli was temporally modulated using a Gaussian temporal function given by $m\left(t\right)=Mexp\;\left\{\;-t^{2}/\left(2\sigma_{t}^{2}\right)\right\}$, where *M* is the peak contrast (0.85 for both experiments) and the temporal standard deviation *σ*_t_ controls the duration of the stimulus, defined as (2 × *σ*_t_).

### Procedure

In each trial of both experiments, during a temporal interval of 500 ms, a small cross (0.25 × 0.25 deg) was presented in the center of the screen (in Experiment 1, two crosses were presented at ± 7.13 deg of eccentricity), and the luminance of the cross was modulated using a Gaussian temporal function with a standard deviation of 80 ms (i.e., changing from gray to black to gray). This fixation cross has the purpose of helping to maintain fixation in the center of the screen before the presentation of the stimuli. After the cross disappeared, the stimuli appeared (drifting leftward or rightward randomly) during a notional presentation interval of 1,000 ms. The contrast of the stimulus was modulated by a Gaussian temporal function, so in general, the stimulus was visible for only a small fraction of the presentation interval. We define the duration of the stimuli as twice the temporal standard deviation (2 × *σ*_t_). After the stimulus disappeared, the participants pressed a button to indicate the direction of motion (left or right). The next trial started after the participant's response. The duration of the stimulus was controlled with a Bayesian adaptive staircase (Treutwein, 1995). The characteristics of the staircase can be seen in Serrano-Pedraza et al. (2013). Duration thresholds are the minimum time needed to discriminate the correct direction of motion (left or right) for a performance of 82% correct responses. The value of the duration threshold was the mean of the final probability distribution (King-Smith, Grigsby, Vingrys, Benes, & Supowit, 1994). Each staircase ended after 40 trials and was interleaved randomly for the small and large window sizes. Three duration thresholds were estimated per spatial window size (small/large) and viewing condition (monocular left, monocular right, and binocular), in total 18 thresholds per subject. No feedback about the correctness of the responses was provided, and practice sessions were performed before starting.

We performed two experiments. In both experiments, we measured duration thresholds for three conditions: binocular, monocular left eye, and monocular right eye. The difference between both experiments was the technique used for achieving monocular viewing. In Experiment 1, we used a mirror stereoscope, and in Experiment 2, we used an eyepatch (see apparatus section).

In Experiment 1, we interleaved viewing conditions starting always with the monocular condition (e.g., monocular, binocular, monocular). In each monocular condition, four thresholds were estimated (small-stimulus left eye, small-stimulus right eye, large-stimulus left eye, and large-stimulus right eye). The four staircases were interleaved randomly, so we mixed eyes and sizes during the session. In the binocular condition, we interleaved two staircases, small stimulus and large stimulus. In total, the two monocular conditions were tested three times (in total 12 thresholds), and the binocular conditions were tested three times (in total 6 thresholds). Thus, 18 thresholds were obtained in total.

In Experiment 2, we also interleaved viewing conditions (monocular left, monocular right, and binocular). In this experiment, we ran nine interleaved sessions: three monocular-left conditions + three monocular-right conditions + three binocular conditions. In each session, we estimated two thresholds (one for large and one for small stimulus), and thus 18 thresholds were obtained in total. In this experiment, participants rested 3 min after each patch removal.

In both experiments, the three different thresholds (in logarithmic units) obtained for each condition/size were averaged for analysis. The order of the sessions can be seen in the data file that can be downloaded from http://www.ucm.es/serranopedrazalab/publications.

### Suppression index

In order to estimate the strength of the psychophysical suppression, we computed a Motion Suppression Index (MSI) (Arranz-Paraiso & Serrano-Pedraza, 2018; Melnick et al., 2013; Read et al., 2015; Tadin et al., 2006; Troche et al., 2018; Yazdani et al., 2015). For each subject, the MSI was estimated by subtracting the duration thresholds (D) in logarithmic units for the large minus the small stimulus. The suppression index MSI was defined as follows:

(1) $$\mathrm{MSI}=\log_{10}\left(D_{\mathrm{large}}\right)-\log_{10}\left(D_{\mathrm{small}}\right),$$

where D_large_ and D_small_ are the duration thresholds for the large and small moving stimulus, respectively.

## Results

### Experiment 1: Comparing duration thresholds for binocular and monocular conditions using a mirror stereoscope

**Figure 2.**
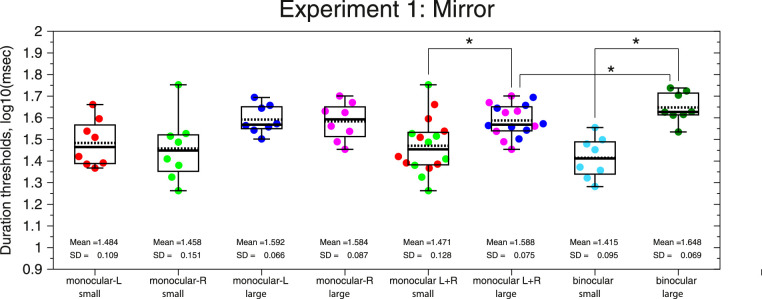
Beeswarm plots and boxplots of the duration thresholds from Experiment 1 (*n* = 8) for small and large stimuli and for binocular and monocular conditions (L, R, and L + R = average of both monocular conditions). The black line inside the boxplot shows the median, and the dotted line shows the mean. The bottom line of the box corresponds to the 25th percentile (Q1) and the upper line to the 75th percentile (Q3). The upper whisker corresponds to the largest value that is less than or equal to Q3 + 1.5 × interquartile range (IQR), and the lower whisker corresponds to the lowest value that is greater than or equal to Q1 – 1.5 × IQR, where IQR = Q3–Q1 is the interquartile range. The asterisk means significant differences after Bonferroni correction (*p* < 0.05/4).

The results from Experiment 1 are shown in Figure 2. Binocular viewing results are consistent with previous studies, for example, Tadin et al. (2003), Melnick et al. (2013), Yazdani et al. (2015), or Arranz-Paraíso and Serrano-Pedraza (2018). That is, duration thresholds for large high-contrast stimuli are nearly twice as high as for small ones (large: dark-green dots; mean ± SD; D_binocular_large_ = 1.648 ± 0.069 log_10_ (ms), 44.9 ± 7.1 ms, *n* = 8; small: cyan dots; mean ± SD; D_binocular_small_ = 1.415 ± 0.095 log_10_ (ms), 26.5 ± 5.8 ms, *n* = 8; repeated-measures *t* test, *t*(7) = 8.417, *p* = 6.58 × 10^−5^, using logarithmic units, *n* = 8).

The results for monocular viewing show the same qualitative effect: at high contrasts, large stimuli have longer duration thresholds than small stimuli. There was no detectable difference between monocular duration thresholds obtained with the left versus right eye, for either small or large stimuli (small: red and green dots, repeated-measures *t* test, *t*(7) = 0.85, *p* = 0.424, using logarithmic units, *n* = 8; large: blue and magenta dots, repeated-measures *t* test, *t*(7) = 0.47, *p* = 0.653), so we combined results from both eyes. Duration thresholds for large stimuli (mean ± SD; D_monocular_large_ = 1.588 ± 0.075 log_10_ (ms), 39.2 ± 6.7 ms, *n* = 16) were significantly higher than duration thresholds for small ones (mean ± SD; D_monocular_small_ = 1.471 ± 0.128 log_10_ (ms), 30.9 ± 9.9 ms, *n* = 16) (repeated-measures *t* test, *t*(15) = 4.838, *p* = 2.17 × 10^−4^, using logarithmic units, *n* = 16).

In order to compare duration thresholds for monocular and binocular viewing, we used a linear mixed-effects model (MATLAB function “fitlme”) fitted by restricted maximum likelihood and assuming this formula: LogThresholds ∼ 1 + Size*Viewing + (1 | ID). The dependent variable was the duration thresholds (log_10_ (ms)); the two fixed effects were size (small/large) and viewing condition (monocular/binocular), and there was one random effect (subject's ID number). The model had four fixed-effects coefficients, including intercept of the model (α_0_ = 1.648; 95% CI, 1.576, 1.719), size condition (β_1_ = −0.233; 95% CI, −0.29, −0.176), viewing condition (β_2_ = −0.06; 95% CI, −0.109, −0.011), and Size × Viewing interaction (β_3_ = 0.116; 95% CI, 0.046, 0.186), and eight random-effects coefficients or random intercepts (i.e., unique intercept for each subject, SD = 0.083; 95% CI, 0.047, 0.145) and random variance or residuals (SD = 0.056; 95% CI, 0.045, 0.071). The deviance of the fitted model was −99.99. Analysis of variance (ANOVA; marginal test with Satterthwaite degrees of freedom, Type III test of fixed effects) shows that there is a highly significant main effect of size (large/small) on duration thresholds (*F*_1,__37_ = 102.54, *p* = 3.25 × 10^−12^). There is no overall main effect of binocularity (monocular/binocular) on duration thresholds (*F*_1,__37_ = 0.015, *p* = 0.903), but the interaction between size and viewing condition is significant (*F*_1,__37_ = 11.26, *p* = 0.0018).

The strength of suppression for each viewing condition shows that the MSI for the binocular condition (mean ± SD; MSI_binocular_ = 0.233 ± 0.078 log_10_ (ms), *n* = 8) is twice the MSI found for monocular viewing condition (mean ± SD; MSI_monocular_ = 0.117 ± 0.097 log_10_ (ms), *n* = 16, left and right eye). Linear mixed-effects model was adjusted using the MATLAB function “fitlme” fitted by restricted maximum likelihood and assuming this formula: MSI ∼ 1 + Viewing + (1 | ID) (deviance = −40.66). ANOVA marginal test with Satterthwaite degrees of freedom (Type III test of fixed effects) shows that there is a highly significant effect of viewing condition (monocular/binocular) on motion suppression (*F*_1,__15_ = 15.3, *p* = 0.0014).

### Experiment 2: Comparing duration thresholds for binocular and monocular conditions using an eyepatch

In Experiment 1, we presented mean luminance to the nonstimulated eye, so we wondered whether this reduced surround suppression under monocular viewing could be generalized to other forms of eye occlusion. Thus, in Experiment 2, a different presentation technique was used. We used an opaque eyepatch and increased the sample to 31 participants.

**Figure 3.**
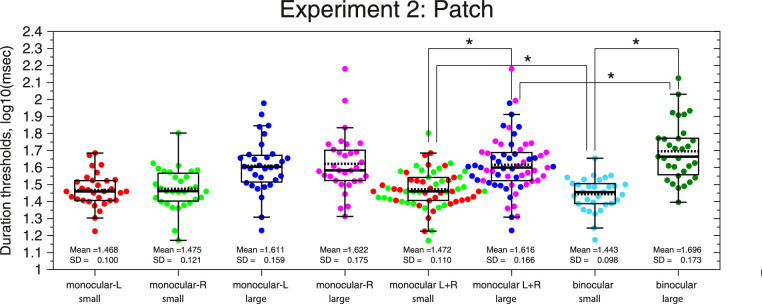
Beeswarm plots and boxplots of the duration thresholds from Experiment 2 (*n* = 31) for small and large stimuli and for binocular and monocular conditions (L, R, and L + R = average of both monocular conditions). Colored dots show logarithmic duration thresholds from individual subjects. The black line inside the boxplot shows the median, and the dotted line shows the mean. The bottom line of the box corresponds to the 25th percentile (Q1) and the upper line to the 75th percentile (Q3). The upper whisker corresponds to the largest value that is less than or equal to Q3 + 1.5 × IQR, and the lower whisker corresponds to the lowest value that is greater than or equal to Q1 – 1.5 × IQR, where IQR = Q3–Q1 is the interquartile range. The asterisk means significant differences after Bonferroni correction (*p* < 0.05/4).

The results from Experiment 2 are shown in Figure 3. As in Experiment 1 and previous studies, duration thresholds for binocularly-viewed large high-contrast stimuli are again nearly twice those for small stimuli (large: dark-green dots; mean ± SD; D_binocular_large_ = 1.696 ± 0.173 log_10_ (ms), 53.9 ± 24.4 ms, *n* = 31; small: cyan dots; mean ± SD; D_binocular_small_ = 1.443 ± 0.098 log_10_ (ms), 28.4 ± 6.2 ms, *n* = 31; repeated-measures *t* test, *t*(30) = 10.351, *p* = 2.04 × 10^−11^, using logarithmic units, *n* = 31).

As we have shown in Experiment 1, under monocular viewing, the difference between duration thresholds using the left or right eye is not significant for either small or large stimuli (small: red and green dots, repeated-measures *t* test, *t*(30) = 0.699, *p* = 0.489, using logarithmic units, *n* = 31; large: blue and magenta dots, repeated-measures *t* test, *t*(30) = 0.782, *p* = 0.44, using logarithmic units, *n* = 31). Thus, for the next analysis, we will combine the results from both eyes. The results for the combined monocular viewing condition follow the same pattern found in the binocular condition; duration thresholds for large stimuli (mean ± SD; D_monocular_large_ = 1.616 ± 0.166 log_10_ (ms), 44.8 ± 21.1 ms, *n* = 62) are significantly higher than duration thresholds for the small ones (mean ± SD; D_monocular_small_ = 1.472 ± 0.11 log_10_ (ms), 30.6 ± 8.1 ms, *n* = 62) (repeated-measures *t* test, *t*(61) = 7.806, *p* = 9.46 × 10^−11^, using logarithmic units, *n* = 62).

As in Experiment 1, in order to compare duration thresholds for monocular and binocular viewing, we fitted a linear mixed-effects model. The model had four fixed-effects coefficients, intercept of the model (α_0_ = 1.696; 95% CI, 1.646, 1.746), size (β_1_ = −0.253; 95% CI, −0.296, −0.209), viewing condition (β_2_ = −0.0798; 95% CI, −0.118, −0.042), and size × viewing interaction (β_3_ = 0.108; 95% CI, 0.055, 0.161), and 31 random-effects coefficients or random intercepts (i.e., unique intercept for each subject, SD = 0.111; 95% CI, 0.084, 0.147) and random variance or residuals (SD = 0.087; 95% CI, 0.078, 0.097). The deviance of the fitted model was −285.86. ANOVA marginal test with Satterthwaite degrees of freedom (Type III test of fixed effects) is similar to Experiment 1. There is a highly significant main effect of size (large/small) on duration thresholds (*F*_1,__152_ = 215.96, *p* = 5.53 × 10^−31^). There is no overall main effect of binocularity (monocular/binocular) on duration thresholds (*F*_1,__152_ = 3.644, *p* = 0.058), but the interaction between size and viewing conditions is highly significant (*F*_1,__152_ = 15.92, *p* = 1.03 × 10^−4^).

The strength of suppression for each viewing condition shows that the MSI for each binocular condition (mean ± SD; MSI_binocular_ = 0.253 ± 0.136 log_10_ (ms), *n* = 31) is 1.75 times higher than the MSI found for the monocular viewing condition (mean ± SD; MSI_monocular_ = 0.145 ± 0.146 log_10_ (ms), *n* = 62, left and right eye). Linear mixed-effects model was adjusted using the MATLAB function “fitlme” fitted by restricted maximum likelihood and assuming this formula: MSI ∼ 1 + Viewing + (1 | ID). We had two fixed-effects coefficients: intercept of the model (α_0_ = 0.145; 95% CI, 0.0976, 0.192) and viewing condition (β_1_ = 0.108; 95% CI, 0.074, 0.142). We also had 31 random-effects coefficients or random intercepts (i.e., unique intercept for each subject, SD = 0.12; 95% CI, 0.09, 0.161) and random variance or residuals (SD = 0.078; 95% CI, 0.066, 0.093). The deviance of the fitted model was −135.25. ANOVA marginal test with Satterthwaite degrees of freedom (Type III test of fixed effects) again shows that there is a highly significant effect of viewing condition (monocular/binocular) on motion suppression (*F*_1,__61_ = 39.304, *p* = 4.1 × 10^−8^).

### Experiments 1 and 2

In general, our duration thresholds for small and large stimuli (for both experiments) were lower than those reported in previous studies (Arranz-Paraíso & Serrano-Pedraza, 2018; Melnick et al., 2013). For example, Melnick et al. (2013) estimated duration thresholds for large (80.3 ± 34.29 ms) and small (39.1 ± 17.5 ms) (*n* = 53) stimuli using Gaussian window diameters of 1.8 and 7.2 deg (the diameter corresponds to two times the standard deviation). However, our results are similar to those obtained by Schallmo et al. (2018; see their Figure 2C) using a similar spatial window. Thus, it is important to note that the main difference with previous studies is that our stimuli were spatially windowed by a Butterworth function. The spatial window used here affects the contrast of the whole stimuli (i.e., this is constant inside the spatial window) and the size (i.e., for the same diameter values, the size of the Gabor patch is almost twice larger than our stimuli), and therefore, these differences in contrast and size possibly explain the differences obtained in duration thresholds.

Our analyses have shown that, in both experiments, duration thresholds for monocular and binocular viewing were not significantly different, but the interaction term between size and viewing conditions was always significant. For each experiment, we have compared duration thresholds for small stimuli (monocular vs. binocular) and for large stimuli (monocular vs. binocular). Given that we have two times more data for monocular (i.e., we have data for left and right eye) than for binocular data, we compare the viewing conditions fitting a linear mixed-effects model by restricted maximum likelihood using the MATLAB function “fitlme” with the following formula: LogThresholds ∼ 1 + Viewing + (1 | ID). In order to simplify the exposition of the analysis, we will show only the values of the fixed-effects coefficients (intercept and viewing condition) and the ANOVA test.

For Experiment 1 and small stimuli, we found the next fixed-effects coefficients: intercept of the model (α_0_ = 1.471; 95% CI, 1.39, 1.56) and viewing condition (β_1_ = −0.06; 95% CI, −0.102, −0.009). ANOVA marginal test with Satterthwaite degrees of freedom (Type III test of fixed effects) shows no significant effect of viewing condition (monocular/binocular) on duration thresholds for small stimuli (*F*_1,__15_ = 6.19, *p* = 0.025; this comparison is not significant after Bonferroni correction—after dividing the alpha level (0.05) by 4, that is the number of comparisons performed; see Figure 2), with binocular thresholds being lower. For large stimuli, we had intercept of the model (α_0_ = 1.587; 95% CI, 1.54, 1.64) and viewing condition (β_1_ = 0.06; 95% CI, 0.026, 0.094). The ANOVA marginal test shows that there is also a significant effect of viewing condition (monocular/binocular) on duration thresholds for large stimuli (*F*_1,__15_ = 13.71, *p* = 0.002), with now binocular thresholds being higher.

For Experiment 2, we found similar results. The analysis of small stimuli shows the next fixed-effects coefficients: intercept of the model (α_0_ = 1.472; 95% CI, 1.43, 1.51) and viewing condition (β_1_ = −0.028; 95% CI, −0.046, −0.011). ANOVA marginal test with Satterthwaite degrees of freedom (Type III test of fixed effects) shows that there is a significant effect of viewing condition (monocular/binocular) on duration thresholds for small stimuli (*F*_1,__61_ = 10.17, *p* = 0.002), with binocular thresholds being lower. Finally, for large stimuli, we had intercept of the model (α_0_ = 1.616; 95% CI, 1.56, 1.67) and viewing condition (β_1_ = 0.08; 95% CI, 0.05, 0.111). The ANOVA marginal test shows that there is also a significant effect of viewing condition (monocular/binocular) on duration thresholds for large stimuli (*F*_1,__61_ = 25.87, *p* = 3.74 × 10^−6^), with binocular thresholds being higher.

These differences in duration thresholds affected the MSI consistently, showing that the MSI is significantly smaller for monocular than for binocular condition in both experiments.

Here, we analyze the MSI of both experiments. Thus, first we fitted by restricted maximum likelihood a linear mixed-effects model using the MATLAB function “fitlme” and assumed this formula: MSI ∼ 1 + Viewing*Experiment + (1 | ID), where we had four fixed-effects coefficients, including intercept of the model (α_0_ = 0.145; 95% CI, 0.1007, 0.1889), viewing condition (β_1_ = 0.108; 95% CI, 0.075, 0.141), experiment (β_2_ = −0.0278; 95% CI, −0.125, −0.069), and Viewing × Experiment interaction (β_3_ = 0.008; 95% CI, −0.065, 0.082), and 39 random-effects coefficients or random intercepts (i.e., unique intercept for each subject, SD = 0.111; 95% CI, 0.086, 0.145) and random variance or residuals (SD = 0.076; 95% CI, 0.065, 0.0895). The deviance of the fitted model was −174.104. ANOVA marginal test with Satterthwaite degrees of freedom (Type III test of fixed effects) shows that there is a highly significant effect of viewing condition (monocular/binocular) on MSI (*F*_1,__76_ = 41.21, *p* = 1.06 × 10^−8^). However, we found no differences in MSI between experiments (*F*_1,__38.26_ = 0.246, *p* = 0.623) and no interaction effect (*F*_1, 76_ = 0.047, *p* = 0.829).

**Figure 4.**
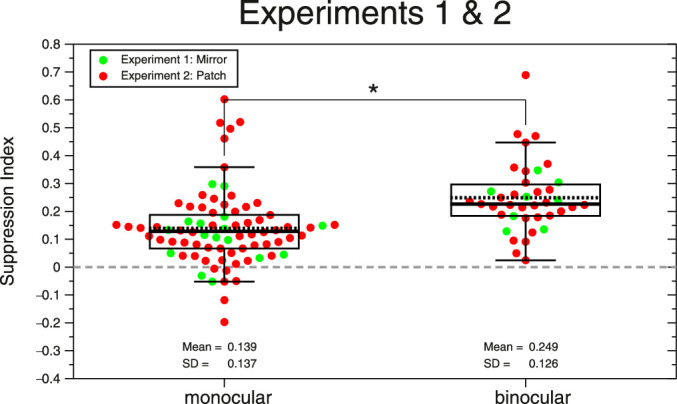
Beeswarm plots and boxplots of the MSI from Experiments 1 and 2 and two viewing conditions: binocular (*n* = 39) and monocular (note that the plot has 78 dots). Each dot shows the suppression index of each participant. Green dots correspond to the MSI from Experiment 1. Red dots are the MSI from Experiment 2. The black line inside the boxplot shows the median, and the dotted line shows the mean. The bottom line of the box corresponds to the 25th percentile (Q1) and the upper line to the 75th percentile (Q3). The upper whisker corresponds to the largest value that is less than or equal to Q3 + 1.5 × IQR, and the lower whisker corresponds to the lowest value that is greater than or equal to Q1 – 1.5 × IQR, where IQR = Q3–Q1 is the interquartile range. The asterisk means significant differences (*p* < 0.05).

Therefore, we combine the MSI of both experiments (see Figure 4)—in green, the MSI from Experiment 1 and, in red, the MSI from Experiment 2. The suppression strength for binocular condition (mean ± SD; MSI_binocular_ = 0.249 ± 0.126 log_10_ (ms), *n* = 39) is 1.79 times higher than the MSI found for the monocular viewing condition (mean ± SD; MSI_monocular_ = 0.139 ± 0.137 log_10_ (ms), *n* = 78, left and right eye).

Finally, using the combined MSI data from both experiments, we fitted by restricted maximum likelihood a linear mixed-effects model using the MATLAB function “fitlme” and assumed this formula: MSI ∼ 1 + Viewing + (1 | ID). We had two fixed-effects coefficients: intercept of the model (α_0_ = 0.139; 95% CI, 0.1, 0.178) and viewing condition (β_1_ = 0.11; 95% CI, −0.14, −0.08). We also had 39 random effects coefficients or random intercepts (i.e., unique intercept for each subject, SD = 0.11; 95% CI, 0.084, 0.143) and random variance or residuals (SD = 0.076; 95% CI, 0.065, 0.089). The deviance of the fitted model was −182.794. ANOVA marginal test with Satterthwaite degrees of freedom (Type III test of fixed effects) shows that there is a highly significant effect of viewing condition (monocular/binocular) on MSI (*F*_1,__77_ = 54.117, *p* = 1.75 × 10^−10^).

### Do different mechanisms mediate monocular versus binocular surround suppression?

In this final analysis, we carry out an individual-differences analysis to ask whether surround suppression under monocular and binocular viewing is driven by the same mechanism. The large differences that we have found in MSI could suggest that different surround suppression mechanisms are activated under monocular and binocular viewing. Although all previous evidences suggest that the psychophysical surround suppression is related to the activation of MT neurons (see introduction), we have not found physiological evidence about different monocular/binocular surround suppression mechanisms in MT. It is interesting to note that different monocular and binocular suppression mechanisms have been found in the striate cortex. For example, Webb et al. (2005) found two mechanisms of surround suppression, in which one is binocularly driven and prominent when the classical receptive field (CRF) is stimulated with high-contrast stimuli (late mechanism) and the other is monocularly driven and prominent when the CRF is stimulated with low-contrast stimuli (early mechanism). Interestingly, recent fMRI studies have found that neural responses in human MT complex (hMT+) reproduce the perceptual suppression and summation at high and low contrast, respectively (Er, Pamir, & Boyaci, 2020; Schallmo et al., 2018). However, opposite results have been found for early visual cortex (EVC) responses. For example, Schallmo et al. (2018) have found that responses in EVC (aggregated activity of V1, V2, and V3) only reproduce perceptual suppression, even for low-contrast stimuli. On the other hand, Er et al. (2020) have found that responses in V1 show strong surround facilitation for both low- and high-contrast stimuli (see discussion in Er et al. [2020] about the differences between both studies).

**Figure 5.**
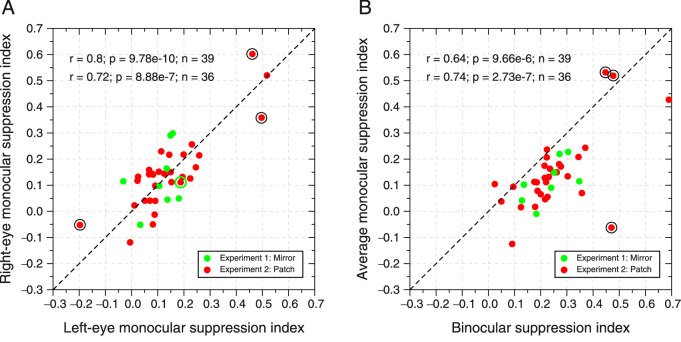
Scatterplots of the MSI. Green dots, MSI from Experiment 1. Red dots, MSI from Experiment 2. (**A**) Right-eye monocular MSI versus left-eye monocular MSI. (**B**) Average monocular MSI (left and right MSI) versus binocular MSI. Two Pearson correlations and *p* values are shown per panel. One is calculated using all data points (*n* = 39) and the other after removing the influential observations (dots with Cook's distance higher than three times the mean of all Cook's distances) (*n* = 36). The dots removed from the correlation are inside the black circles. The dashed line represents the identity line. Note that in panel B, almost all dots are shifted below the identity line.

Here, we are going to apply the same logic used to study other visual mechanisms (Billock & Harding, 1996; Peterzell & Teller, 1996; Sekuler, Wilson, & Owsley, 1984; Yazdani et al., 2015); for example, if two different stimuli are detected by the same visual mechanism, then the correlation between the detection thresholds for both stimuli should be higher than if the stimuli were detected by different mechanisms. Thus, the logic behind our analysis is that if MSI for monocular and binocular shows similar correlation to monocular-left versus monocular-right, then this would suggest the same surround suppression mechanism is underlying both types of viewing. On the other hand, if the correlation between monocular viewing conditions (left vs. right) is much higher than monocular versus binocular, then this would suggest different surround mechanisms for monocular and binocular viewing. The scatterplots of the MSI of both experiments are shown in Figure 5. Pearson correlation between right-eye monocular MSI and left-eye monocular MSI was *r* = 0.80, 95% CI, 0.65, 0.89, *p* = 9.78 × 10^−10^ (*n* = 39). The correlation between the averaged monocular (left/right) MSI and the binocular MSI was *r* = 0.64, 95% CI, 0.41, 0.8, *p* = 9.66 × 10^−6^ (*n* = 39).

Thus, the correlation between monocular MSI is higher than between monocular and binocular, although the difference is marginally not significant. However, influential observations could be affecting the correlations. To find out whether there are any highly influential observations in our sample, we computed Cook's distance. We then removed the observations with Cook's distance higher than three times the mean of all Cook's distances. We found three observations for each correlation (one subject was common in both correlations) that are plotted inside the black circles in Figures 5A,B.

After removing the influential observations, the correlations were very similar between viewing conditions. In particular, the correlation between right-eye monocular MSI and left-eye monocular MSI was *r* = 0.72, 95% CI, 0.51, 0.85, *p* = 8.88 × 10^−7^ (*n* = 36), and that between the averaged monocular (left/right) MSI and the binocular MSI was *r* = 0.74, 95% CI, 0.54, 0.86, *p* = 2.73 × 10^−7^ (*n* = 36). Therefore, our analysis does not support the hypothesis of different surround suppression mechanisms for monocular and binocular viewing.

## Discussion

We have presented the results of two experiments in which we have measured duration thresholds for small and large drifting gratings under two viewing conditions (monocular vs. binocular) and two different stimulus presentation techniques (mirror stereoscope vs. eyepatch). In both experiments, we have found consistent results. First, duration thresholds for large stimuli were higher than duration thresholds for small stimuli (Experiment 1: ratio between large/small durations in ms was 1.7 for binocular and 1.27 for monocular; Experiment 2: 1.9 for binocular and 1.46 for monocular), replicating previous results (Lappin et al., 2009; Serrano-Pedraza, Hogg, & Read, 2011; Serrano-Pedraza et al., 2013; Tadin et al., 2003; Tadin et al., 2019; Tadin & Lappin, 2005). Second, in both experiments, we have found that duration thresholds for large stimuli were higher for binocular than for monocular viewing conditions, and duration thresholds for small stimuli were lower for binocular than for monocular viewing conditions. These differences have an important effect when combined into the MSI. In fact, in both experiments, the MSI obtained under monocular conditions was significantly lower than for the binocular condition. Combining the MSI of both experiments, we have found that the MSI for the binocular condition was 1.79 times higher than the MSI for the monocular condition. Therefore, surround suppression is reduced under monocular viewing independently of the technique used to present the stimulus in one eye (mirror stereoscope vs. eyepatch). This difference is much stronger than previous research that has compared monocular versus binocular suppression (Paffen et al., 2005). The study by Paffen et al. (2005) and the present one differ in many aspects, but the main difference is that they measure depth of binocular rivalry suppression, and we measure duration thresholds. Also, both eyes, independently of the viewing condition, were stimulated in fovea along their experiments, with a moving grating in one eye and a static concentric ring in the other eye. In our monocular condition, only one eye was stimulated.

We also have found that the correlation between monocular left-eye MSI and monocular right-eye MSI is similar to the correlation between the averaged monocular left/right MSI versus binocular MSI. This suggests that the same surround suppression mechanism is active during both monocular and binocular viewing but has a greater effect during binocular viewing.

In Experiment 2, we used an eyepatch to perform the monocular conditions. It is well known that short-term monocular deprivations have an effect on visual processing (Lunghi, Burr, & Morrone, 2011, 2013; Lunghi et al., 2015; Serrano-Pedraza et al., 2015; Wang, McGraw, & Ledgeway, 2020; Zhou et al., 2015). In those studies, they usually occlude one eye using a patch for 2.5 hr, and in our Experiment 2, we occluded the eye for 2 min (that was the time needed to obtain two thresholds in each session). However, in a recent study using shorter deprivation periods of 3 to 6 min, Han et al. (2020) found that the deprived eye changed from spatial surround suppression before deprivation to surround facilitation afterward; no binocular conditions were tested. This raises the question of whether the lower suppression index we found in the monocular condition might reflect a similar process, with the monocular results being affected by a shift toward facilitation. We think this is unlikely for two reasons. First, in Experiment 1, using a mirror stereoscope, we randomly interleaved left-eye and right-eye trials, so neither eye was deprived. We still obtained a significant effect of viewing condition, just as in Experiment 2, in which one eye was deprived for 2 min with a patch. Second, in Experiment 2, we allowed a recovery period of 3 min in between monocular sessions. Han et al. (2020) found their effect immediately after deprivation, while Kim et al. (2017) found that the effect of a 15-min deprivation had largely decayed back to baseline after 5 min of recovery. Nevertheless, we examined our data to see if we could detect any effect attributable to previous deprivation rather than monocular viewing per se. In Experiment 2, we interleaved monocular and binocular conditions when measuring the total of 18 thresholds, so we cannot use the final thresholds to examine any effect of temporal order. In 8 out of our 31 participants, the first monocular run happened to be in the right eye and followed immediately by a run in the left eye (each run consisting of interleaved staircases measuring thresholds for large and small stimuli). This means that the right-eye measurement was in an undeprived eye, while the left-eye measurement was in an eye that had been deprived for 2 min. We can therefore use these first threshold measurements to look for an effect of deprivation. If deprivation had altered surround suppression, we should see a difference between thresholds in the two eyes. In fact, repeated-measures *t* test showed no significant differences for either small or large stimuli (right-eye small vs. left-eye small, *t*(7) = 0.303, *p* = 0.771; right-eye large vs. left-eye large, *t*(7) = –0.058, *p* = 0.955). In another recent study, Kim, Kim, and Blake (2017) show that “depriving” an eye of input for 15 min via continuous flash suppression (CFS) temporarily imbalances ocular dominance in a similar way to that obtained by physical patching for 15 min. The effect found using CFS means that there is no need to use a patch to create an imbalance in ocular dominance, and that could be applicable to our Experiment 1. However, once again, the way we obtained the thresholds interleaving eyes and sizes in the same session (i.e., interleaving randomly the trials of each staircase, so in each trial during the monocular session, the left or right eye could be stimulated with a small or large stimulus) means that there is no monocular deprivation during the testing, and therefore our results cannot be explained by an imbalance in ocular dominance.

Another potential explanation of our results could be that under monocular viewing, the perceived contrast of the stimulus is reduced, and as a consequence, duration thresholds for larger stimuli are reduced, and duration thresholds for small stimuli increase, as previously shown (Serrano-Pedraza et al., 2011; Tadin et al., 2003). Therefore, the MSI (the difference between large and small log duration threshold) is smaller. From the results of Tadin et al. (2003), a reduction of 1.79 in MSI would require approximately a contrast reduction from 92% to 22%, that is, around 4.2 times contrast reduction. It is well known that there is an advantage of binocular vision in spatial vision. In particular, detection thresholds under binocular viewing are $\sqrt{2}$ times lower than under monocular viewing (Campbell & Green, 1965; Legge, 1984). Recent studies, however, have shown that the ratio is about 1.7, suggesting a reduced contrast perception under monocular viewing (Baker et al., 2007; Meese et al., 2006; Meese & Baker, 2011; Rose, 1980). However, this advantage of binocular vision in spatial vision is present only for contrasts lower than 20%, which is much lower than the contrast used in our experiments (85%). It therefore seems very unlikely that our results can be explained by a reduction in effective contrast for the monocular stimuli. In a different study, comparing monocular and binocular processing in three global motion tasks, Hess et al. (2007) found that there is a binocular advantage (i.e., lower global motion coherence thresholds) that depends on the contrast of the stimuli. Interestingly, when the contrast was higher than 0.1, their results showed no difference between monocular and binocular conditions. However, it is important to note that previous studies did not present the stimulus for durations as short as 30 to 50 ms as in the present study. Therefore, we cannot rule out that under monocular viewing and short durations, there is a reduction in perceived contrast. Matching-contrast experiments for short presentation durations would be needed to rule out this hypothesis.

Previous results have found that suppressive center-surround interactions increase with increasing speed (Lappin et al., 2009), so a second potential explanation is that perceived speed under monocular viewing would be lower than under binocular viewing, and therefore, this reduced speed perception could reduce the MSI. However, no difference has been found between the perceived speed of monocular- and binocular-viewed grating stimuli over a wide range of speeds (Thompson, 2006).

**Figure 6.**
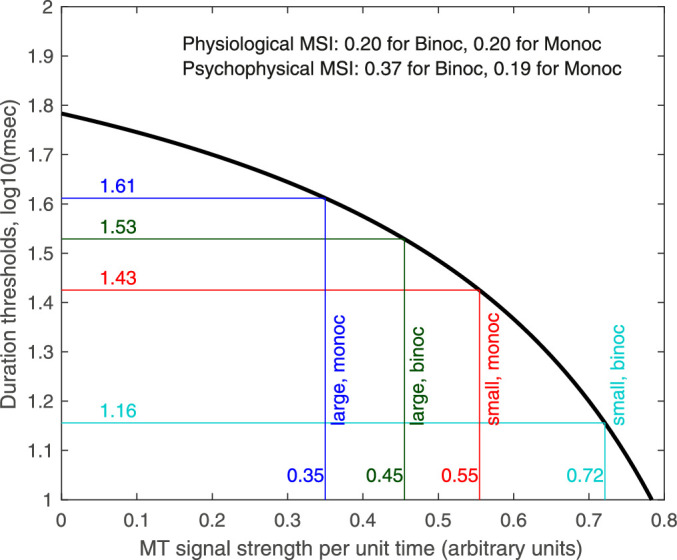
The black line shows a hypothetical linking function relating MT activity to log duration thresholds. Stronger activity results in lower thresholds, but this relationship becomes steeper as MT activity increases toward maximum. The colored lines mark the four stimulus conditions. For our hypothetical MT, large stimuli elicit only 63% of the activity of small stimuli, giving a physiological surround suppression index of log_10_(Signal_small_/Signal_large_) = log_10_(0.72/0.45) = 0.2 and log_10_(0.55/0.35) = 0.2 for both binocular and monocular stimuli, respectively. However, the nonlinear linking function results in greater psychophysical surround suppression for binocular: log_10_(T_large_/T_small_) = 1.529 – 1.156 = 0.37 for binocular versus 1.612 – 1.425 = 0.19 for monocular. This demonstrates how psychophysical surround suppression can depend on viewing condition (monocular vs. binocular) even if the physiological surround suppression does not. Note, though, that a constant physiological suppression index cannot explain why binocular thresholds are higher than monocular for large stimuli, as we observe.

Turning to physiology, the existing work documenting monocular versus binocular responses of MT neurons is not sufficient to account fully for our findings. Most MT neurons are activated more strongly by binocular than monocular stimuli, predicting shorter-duration thresholds for binocular stimuli. We did observe this for the small stimuli, but for large stimuli, thresholds were actually longer for binocular stimuli. This implies that the binocular/monocular ratio of the MT neurons supporting perception must change depending on stimulus size. If this were not the case, we might still observe a difference in psychophysical surround suppression for monocular versus binocular, depending on the function linking neural activity to perceptual thresholds (about linking propositions, see Teller, 1984). For example, if the linking function became steeper with increasing neural activity over the relevant range, the increased response for binocular stimuli could cause an increased psychophysical suppression index, even if the physiological suppression index were the same as for monocular (see Figure 6). However, a monotonic linking function can explain why duration thresholds for small stimuli are shorter for binocular than monocular viewing, but it cannot explain why binocular thresholds are higher than monocular thresholds for large stimuli. That finding, if it turns out to be reliable, would imply that, over the relevant neuronal populations, physiological surround suppression is greater for binocular than monocular stimuli.

One way for this to occur is if individual neurons showed stronger surround suppression for binocular stimuli. This could occur if suppression from the surround is combined supralinearly across eyes, so it is enhanced if it occurs in both eyes simultaneously. This has not yet been demonstrated. So far, the more complex interactions demonstrated in MT tend to be monocular rather than binocular: For example, component gratings are combined into pattern motion if presented in the same eye but not if presented dichoptically (Tailby et al., 2010). This is consistent with psychophysical work showing a strong monocular component in early motion processing (Alais et al., 1996; Grunewald, 2004; Swanston et al., 1990; Wade et al., 1993;), probably because motion in depth and vergence eye movements mean that retinal motion can be very different in the two eyes.

The output of this early local, monocular motion processing seems to be pooled into large, binocular MT receptive fields (Baker & Bair, 2016; Majaj et al., 2007; Tailby et al., 2010). Especially in neurons tuned to frontoparallel stimuli, binocular combination in MT so far seems rather linear, with 67% of variance in the response to binocular grating explained simply by summing the responses observed to monocular gratings (64% across all neurons; Czuba et al., 2014). Monocular processing followed by linear binocular combination cannot produce stronger surround suppression for binocular stimuli.

However, our psychophysical results could reflect differential activation of neural populations with different suppression strengths. It is quite likely that monocular and binocular stimuli activate different neural subpopulations within MT. A leftward-moving monocular stimulus should weakly activate all MT cells tuned to leftward motion in that eye. The same stimulus presented binocularly should produce similar, weak activation in the subpopulation tuned to motion toward/away from the observer but much stronger activation in the subpopulation tuned to frontoparallel motion (see Czuba et al., 2014, their Figure 2). Thus, binocular surround suppression may reflect mainly physiological surround suppression in MT neurons tuned to frontoparallel motion, while monocular surround suppression may reflect something closer to the average across all MT neurons. If surround suppression is generally stronger in frontoparallel-tuned neurons, this could account for our psychophysical results, even if no individual neuron showed a difference in its surround suppression index for monocular vs.versus binocular stimuli. Our correlation analysis did not provide support for the idea that monocular and binocular stimuli activated different (although overlapping) populations but equally cannot disprove it.

To our knowledge, no one has yet compared binocular versus monocular physiological surround suppression in MT or elsewhere. Such experiments will be an important test of the theory that psychophysical surround suppression does indeed reflect the properties of MT neurons. They will also help to unpack the extent to which the relevant properties are generated in MT versus inherited from earlier areas.

We would like to highlight that our results show the importance of testing both eyes to measure the strength of surround suppression using MSI. Different studies have assumed that the neural strength of the suppressive center-surround interactions could be indirectly inferred through MSI (Tadin, 2015). Other studies have found a link between the MSI and clinical conditions, such as schizophrenia (Tadin et al., 2006), depression (Golomb et al., 2009), aging (Betts et al., 2005; Betts et al., 2009; Tadin & Blake, 2005), epilepsy (Yazdani et al., 2017), autism (Foss-Feig et al., 2013), and even intelligence (Arranz-Paraíso & Serrano-Pedraza, 2018; Melnick et al., 2013). It is interesting to note that in those studies, participants were tested binocularly, but only Arranz-Paraíso and Serrano-Pedraza (2018) tested the spatial visual acuity of each eye and also measured their stereovision. We believe that in some special populations, it would be important to test the visual acuity of each eye independently and also test stereovision, because abnormalities in binocular vision caused by strabismus or other pathologies could explain the inconsistency between previous results and recent studies in depression or autism (Norton et al., 2016; Schauder et al., 2017). For example, the rate of strabismus in autistic (Kaplan et al., 1999) and schizophrenic populations (Agarwal et al., 2017; Ndlovu et al., 2011; Toyota et al., 2004) is higher than in the normal population. Moreover, some studies found a higher rate of strabismus in people with epilepsy (Jensen & Seedorff, 1976; Millar, 1965). Also, aging seems to predispose people to incomitant strabismus (Clark & Demer, 2002). Thus, reduced MSI in some people could be related to the use of only one eye when performing the motion task.

Finally, in a recent study, Tadin et al. (2019) have found a strong link between surround suppression and motion segregation (segregation of moving objects from their background). Thus, our results predict that motion segregation under monocular viewing will be impaired with regard to binocular viewing. Future research would be needed to test this prediction.
